# Clinical implementation of axial angulation of incisors in the course of routine fixed appliance treatment — a retrospective cohort study

**DOI:** 10.1007/s00784-022-04781-7

**Published:** 2022-12-01

**Authors:** Ramtin Davoudi Pour, Spyridon N. Papageorgiou, Sema Safi, Oliver-Steffen Eble, Andreas Jäger, Lina Gölz

**Affiliations:** 1 Department of Orthodontics, University Hospital of Bonn, Bonn, Germany; 2 Clinic of Orthodontics and Pediatric Dentistry, Center of Dental Medicine, University of Zurich, Zurich, Switzerland; 3 Orthodontic Practice, Duisburg, Germany; 4 Department of Orthodontics and Orofacial Orthopedics Friedrich-Alexander, University Erlangen-Nuremberg, Erlangen, Germany

**Keywords:** Crown torque, Multibracket treatment, Clinical study, Angle class, Extraction therapy, Craniofacial configuration, Torque loss, Slot play

## Abstract

**Purpose:**

To identify clinically relevant factors for changes in axial angulation of incisors during routine fixed appliance orthodontic treatment.

**Methods:**

A total of 106 patients (grades 1–2 of IOTN, 64 females, 42 males; mean age: 15.5 years) from a private practice and treated with metal or ceramic brackets were included in this retrospective cohort study. The axial angulation of the upper and lower incisors was measured on lateral cephalograms before insertion of the first rectangular 0.016 × 0.022-in NiTi archwire (T0) and at the end of treatment about 8 weeks after insertion of the working 0.019 × 0.025-in stainless steel archwire (T1). Treatment-related changes according to bracket type, initial situation, premolar extraction, angle class, and skeletal vertical configuration were analyzed.

**Results:**

Although statistically significant treatment-related changes were seen for both the upper incisors (+ 1.3°) and the lower incisors (− 5.2°), only in ten patients (9.4%) was the prescribed torque value of 17° for the upper incisors and in no patient for the lower incisors achieved. A negative association between the induced change of axial angulation of incisors and the initial values was detected for the upper incisors as well as for the lower incisors. A comparison of the angle classes revealed significant differences in incisor changes. At the end of therapy, only a slight change for the upper central incisors in patients in angle class I cases and a significantly greater change in patients with angle class II/2 was observed. Cases with premolar extraction ended with lower axial angulation of the incisor than cases without extraction. The individual analysis of possible influencing factors also revealed an association with the vertical skeletal configuration.

**Conclusions:**

For the first time, the presented data show clinically relevant influencing factors for incisor axial angulation changes of the upper and lower incisors in relation to the torque value of the applied brackets in the course of routine clinical practice. For the orthodontist, it remains mandatory to decide whether a customized system must be individualized in order to achieve individual therapy goals.

## Introduction

In clinical orthodontics, labio-lingual axial angulation of both posterior and anterior teeth is considered to be most important to establish a proper occlusal relationship, an esthetic treatment result and subsequently long-term stability of the orthodontic treatment result. In order to correct axial misalignment, e.g., in the area of the anterior teeth, torque can be transmitted to teeth through brackets using rectangular wires. The generation of “active torque” involves a twist between the wire and the bracket slot. In a strict sense, torque movement in orthodontics is defined as the movement of the root either buccally or lingually. On the other hand, a change of axial angulation of the teeth can result from changes of the root as well as of the crown position [39, 42]. Multiple tools and analytic procedures have been suggested to practically measure the axial angulation of teeth or changes of the same. These are very often difficult to compare because of the measurement devices, and even the chosen reference planes differ. Cephalometric angular measurements, measurements intraorally or on dental casts (e.g., tooth inclination protractor; TIP; MBI, Newport, UK), and analysis of digitized models or intraoral scans have been performed [4, 12, 13, 24, 31, 45].

In the straight-wire technique (SW), to achieve torque movement of the incisors, a rectangular archwire with appropriate dimensions for torque is tied into the bracket slot which has a predefined orientation on the tooth surface. Since the introduction of the straight wire appliance (SWA) in the 1970s by Andrews [2], there have been many suggested modifications of torque values to be used in pre-adjusted edgewise appliances. But, despite the abundance of research, until today, there is high variability among various prescriptions with respect to incisor torque values. As an example, incisor torque values for pre-adjusted appliances for the maxillary centrals range from 12° in the Roth prescription to 22° in the bioprogressive prescription [50].

**Fig. 1:**
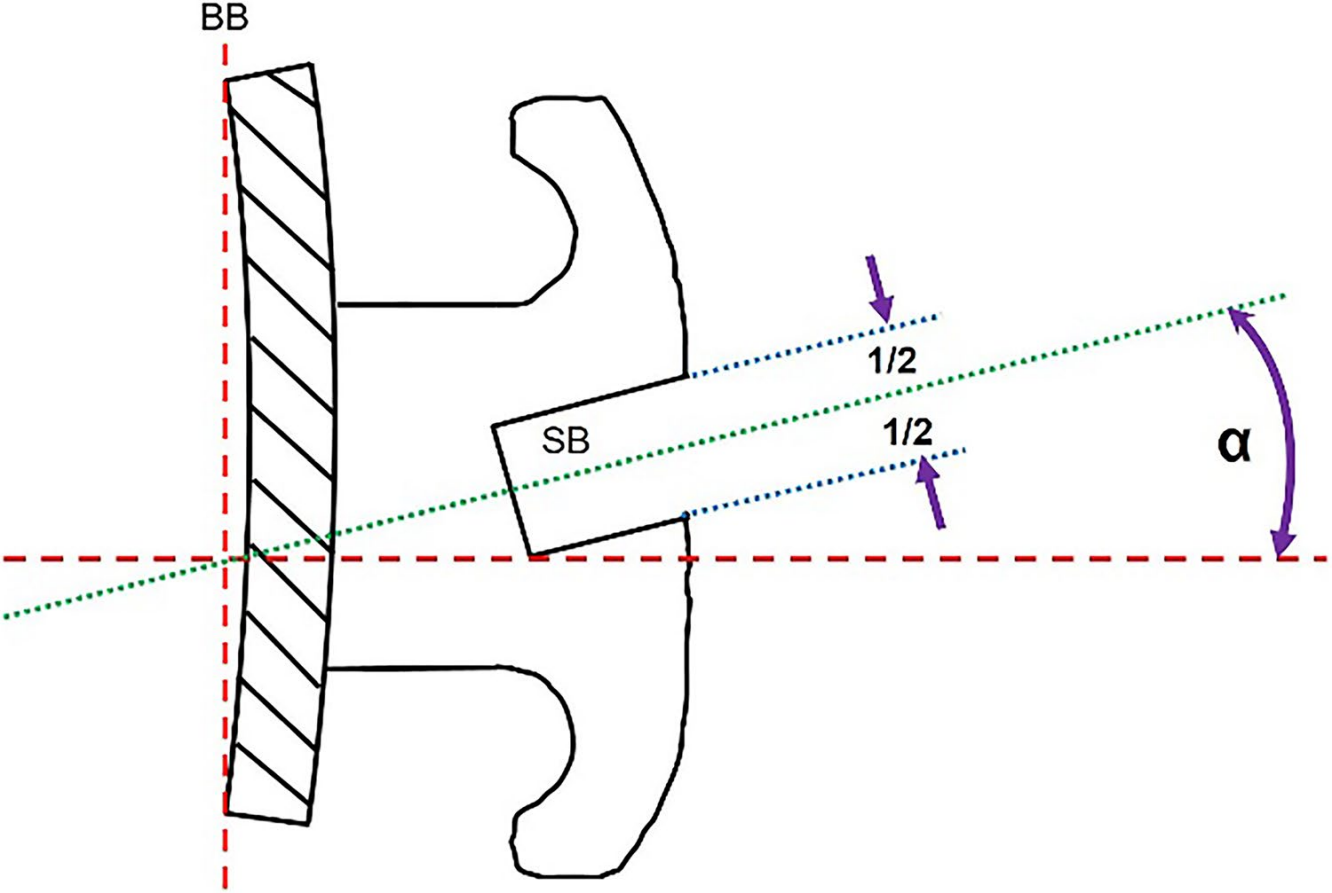
Torque angle in the bracket according to the DIN EN ISO 27020. It is defined as the angle (*α*) between a perpendicular to the bracket base (BB) and a slot bisector (SB)

Technically, according to DIN EN ISO 27020:2010, the torque angle in the bracket is defined as the angle between a perpendicular to the bracket base and a slot bisector (Fig. 1). In theory, at the end of treatment, this angle should correspond with the angle between the bracket base and the archwire plane. For the definite transmission of torque in daily clinical practice, the individual interplay between the form and dimension of the slot and the arch play a crucial role. With respect to the used archwires, conventional wires often show rounded or blurred edges [40]. The more the edges of the arches are rounded, the more torque is lost. Another considerable loss of torque transmission occurs when using non-slot-filling arches as it is common for most routine orthodontic treatment techniques [5, 16, 26–28]. The reason for this is that large slot-filling wires may result in patient discomfort and difficulty in inserting the wires into the slot. In addition, moving teeth along a slot-filling wire may be inhibited by excessive friction. Thus, for most orthodontists using a 0.022″ slot, the working-wire of choice is a 0.019 × 0.025-in stainless steel wire. This provides a compromise between a larger wire that would be difficult to place and would not slide well and a smaller one that would provide less tooth control and would show possible deflection when activated for space closure.

Due to the mentioned “torque play,” a large part of the torque information (nominal torque) introduced into the bracket is lost and a difference to the effective torque results. For the most common bracket-wire combinations, the effective torque can be calculated using a formula published by Gioka and Eliades [16]. In reality, torque play can be determined by different measuring methods. Some studies have investigated torque transmission to the incisors in vitro and by means of finite element studies. The identified influencing factors determined here were as follows: tooth morphology and position of the bracket on the tooth, prescription, bracket type and the type of archwire ligation, torque play in the slot, dimension of the square wire, edge bevel, type of alloy, deformability of the bracket, and interbracket distance [15–17, 19, 30, 36].

Owing to the mentioned complexity of influences, it is nearly impossible to precisely estimate the transmission of the prescription of a specific bracket torque value in the individual clinical situation. Thus, multiple authors performed clinical trials investigating the performance of different bracket-wire-combinations [9, 27, 28, 34, 38, 48].

It was the aim of the present study to investigate the extent to which the initial morphology and therapy measures were relevant for the change of axial angulation of upper and lower incisors in all available patients from an orthodontic practice treated within a defined period of time using lateral cephalograms. The primary goal was to determine the degree to which the nominally prescribed torque values were actually achieved as a change in axial angulation at the end of therapy.

## Material and methods

The presented clinical trial was designed as a retrospective cohort study. Reporting about the study was performed in compliance with “Strengthening the Reporting of Observational studies in Epidemiology (STROBE)” [46]. All patients or their legal guardians were asked and gave their permission to retrospectively analyze their clinical data for anonymous quality assurance purposes.

### Study sample

The final study included a total of 106 patients (42 male/64 female). Baseline characteristics of the patients are presented in Table 1. All of them were treated during the years 2014 until 2016 with a fixed, pre-programmed appliance in a private orthodontic practice. The age of the patients at the start of treatment ranged between 12 and 16 years (mean 15.5 years). All patients treated within the abovementioned time period and who met the following inclusion criteria were included: (i) patients subjected to fixed appliance orthodontic treatment, (ii) adherence of patients to treatment dates and instructions, and (iii) availability of lateral cephalograms before and after insertion of rectangular archwires. The exclusion criteria were as follows: (i) previous orthodontic treatment, (ii) craniofacial anomalies or syndromes, and (iii), aplasia of teeth.

**Table 1:** Baseline characteristics of the 106 patients included in this study

Factor	Category	Data
Gender	Male, *n* (%)	42 (40%)
	Female, *n* (%)	64 (60%)
Age (years)	Mean (SD) [range]	15.5 (2.3) [12.2, 26.9]
Face type	Mesofacial, *n* (%)	39 (37%)
	Brachyfacial, *n* (%)	52 (49%)
	Dolichofacial, *n* (%)	15 (14%)
Race	Caucasian, *n* (%)	53 (50%)
	Oriental, *n* (%)	51 (48%)
	Japanese, *n* (%)	2 (2%)
Angle	Class I, *n* (%)	23 (22%)
	Class II/1, *n* (%)	19 (18%)
	Class II/2, *n* (%)	28 (26%)
	Class III, *n* (%)	36 (34%)
Bracket	Metal, *n* (%)	54 (51%)
	Ceramic, *n* (%)	52 (49%)
Extractions	No, *n* (%)	85 (80%)
	Yes, *n* (%)	21 (20%)
Time T0–T1 (days)	Mean (SD) [range]	272.6 (78.6) [160.0, 570.0]

*SD*, standard deviation

In the course of the study, one patient had to be excluded because of a change of residence and in one patient treatment was discontinued because of insufficient oral hygiene.

### Orthodontic treatment

All patients were treated by two experienced clinicians following the same treatment protocols and were complied with grades 1–2 of IOTN (Index of Orthodontic Treatment Need) [8] upon completion of therapy. For treatment, 54 patients received conventional metal brackets (Victory—3 M Unitek) while 52 patients were treated with self-ligating ceramic brackets (In-Ovation C—GAC). The prescription of the appliance used was MBT in a 22″ slot system. The pre-programmed torque values for the upper and lower central incisors were + 17° and − 6°, respectively.

The brackets were applied following guidelines of McLaughlin and Bennett [26]. In the course of treatment, all patients received treatment wires in the same order: after an initial leveling phase with round wires (0.012-in Ni–Ti, 0.014-in Ni–Ti, 0.016-in Ni-T), different rectangular wires (0.016 × 0.022-in Ni–Ti, 0.017 × 0.025-in Ni–Ti, 0.019 × 0.025-in Ni–Ti, 0.019 × 0.019-in stainless steel, 0.019 × 0.025-in stainless steel) were applied. Appliances were routinely adjusted at an interval of 6–8 weeks. All appliances were ligated using conventional elastomeric ligation or stainless steel ligatures. If indicated, 3/16 intermaxillary elastics with medium force 3.5 oz Class II, Class III were used bilaterally at the time period of rectangular steel wire treatment. Extraction spaces were closed using sliding mechanics with closed coil springs or elastomeric chains. Mean duration of treatment was 18 months.

### Cephalometric analysis

The cephalograms were taken with the same x-ray machine (Orthophos SL Dentsply Sirona) as part of the routine orthodontic progress diagnostics. At time T0, a cephalogram was taken before insertion of the first rectangular arch (0.016 × 0.022-in NiTi) and another cephalogram (T1) at the end of active treatment (final archwire: 0.019 × 0.025-in stainless steel for approximately 8 weeks). The average time between T0 and T1 amounted to 38.94 weeks (range 22.86 to 81.43 weeks). The cephalometric analyses were performed by one single experienced investigator (D.R.) using the OnyxCeph® software.

### Analysis of incisor’s axial angulation

**Fig. 2:**
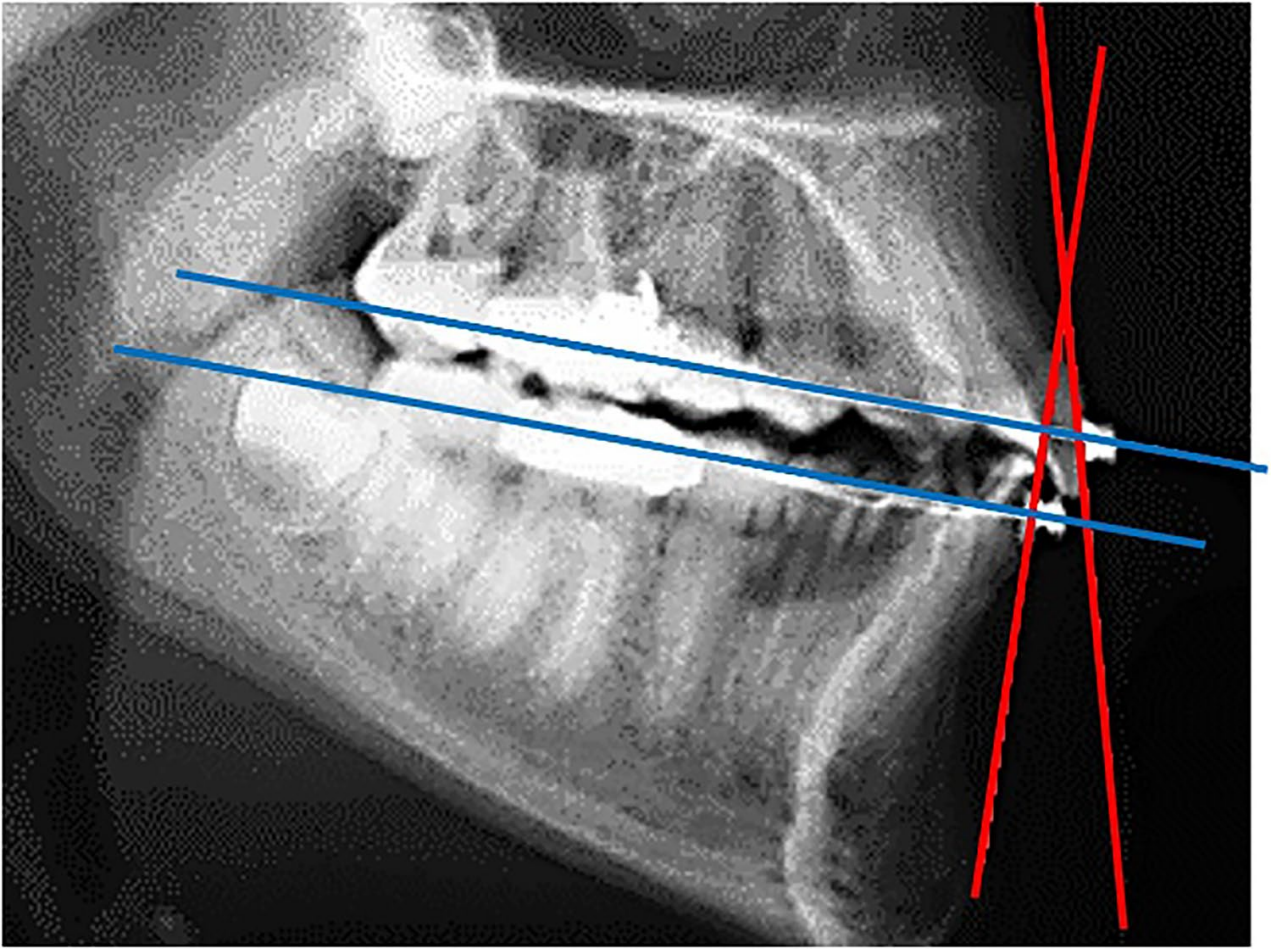
Axial angulations of upper and lower incisors were assessed between a perpendicular to the archwire plane and a line representing the bracket base of the mostly anterior positioned incisors

Instead of using conventional cephalometric angular or linear measurements, analysis of axial angulations of upper and lower incisors was assessed separately for the upper and lower teeth between a perpendicular to the archwire plane and a line representing the bracket base of the mostly anterior positioned incisors (Fig. 2). The archwire planes were established by drawing a line connecting the center of all brackets and representing the course of the upper and lower treatment wires in place. In case of a minor persisting curve of the archwire, the mostly anterior and posterior brackets were chosen.

The cephalograms were also used to evaluate the vertical skeletal configuration (“facial type”: meso-, brachy-, and dolichofacial) by measuring the NL/ML angle. Patients with a mandibular plane angle < 20° were classified as “horizontal” (brachyfacial) whereas patients with a mandibular plane angle > 28° were classified as “vertical” (dolichofacial) [44].

In addition to the determination of the axial angulation of the upper and lower central incisors, the following clinical and therapeutic parameters were recorded: angle classification, craniofacial morphology, bracket type, incorporation of premolar extractions in the treatment plan.

### Intra-examiner reliability

To determine the reproducibility of the measurements, the cephalometric analysis was performed by the blinded examiner on 2 different days. The statistical measuring error for the axial angulation of the incisors values was determined using the Dahlberg formula [20] and was determined to be ± 0.91° for the upper incisors and ± 0.98° for the lower incisors.

### Blinding

Placing orthodontic appliances precludes blinding of the participant. We therefore performed blinding of the investigator analyzing the cephalograms and the person in charge of the statistical analysis of the data, both of them being unaware of the treatment received by each patient.

### Statistical analysis

After checking the normality of the data through graphical inspection and the Shapiro–Wilk test, descriptive statistics were calculated including means and standard deviation (SD). Differences between T0 and T1 or between different subgroups were tested with generalized linear regression modelling and expressed as unstandardized regression coefficients and their 95% confidence interval (CI). Statistical significance was set at a two-tailed *p*-value ≤ 0.05. All analyses were done in Stata 14.0 (StataCorp, College Station, TX) and the study’s dataset is openly provided [14].

## Results

At the time of the design of the trial in a private praxis, no a priori calculation of sample size had been conducted. Instead, we carried out a post hoc power calculations based on the actual data from the primary aim of the study, which was the analysis of the deviation of the achieved axial angulation of the upper and lower incisors from their respective nominal torque values. The measured values at T1 were 4.77° (SD = 3.54°) for the upper incisors and − 6.21° (SD = 4.53°) for the lower ones. Accordingly, the standardized differences (mean difference/standard deviation) can be calculated to be 1.35 and − 1.37 respectively. Using the Altman nomogram [47] for a sample size of *n* = 106 and a significance level of 0.05, the power for both measurements reached a level of 1.0.

Baseline (T0) and follow-up (T1) values for all radiographic measurements together with the changes T1–T0 (delta) are presented in Table 2. Table 3 shows the effect of various factors on treatment-related changes, and Tables 3 and 4 show the mean axial angulation changes for incisors of each jaw separately. Interestingly, the programmed torque of 17° in the maxilla was only observed in ten patients (9.4%) at T1. For the mandibular incisors, the preprogrammed torque of − 6° was not detected in any case. The deficient axial angulation for the upper and lower incisors can also be found in Table 2. For the upper incisors, the deficit between the prescription (Upper centrals: + 17°) and the achieved angulation was 4.77° and for the lower incisors − 6.21° (prescription of lower centrals: − 6°). This means that at the mean, the upper incisors ended up with a more upright and the lower ones with a more proclined angulation than what would have been expected from the prescription. Nevertheless, in absolute terms, more axial angulation change was transferred towards the prescription in the mandible than in the maxilla.

**Table 2:** Baseline (T0) and follow-up (T1) values for all radiographic measurements together with the changes T1–T0 (delta)

	T0		T1		Delta (T1–T0)		
	*n*	Mean (SD) [range]	*n*	Mean (SD) [range]	*n*	Mean (95% *CI*)	*P*
SNA	106	80.62 (4.17) [71.20, 94.10]	106	80.24 (4.33) [69.20, 93.30]	106	− 0.38 (− 0.72, − 0.05)	0.03
SNB	106	77.43 (4.16) [65.20, 93.10]	106	77.51 (4.24) [64.7, 91.6]	106	0.08 (− 0.25, 0.40)	0.65
ANB	106	3.20 (2.20) [− 1.80, 9.70]	106	2.88 (2.36) [− 3.30, 8.80]	106	− 0.33 (− 0.55, − 0.10)	0.004
WITS	106	1.63 (2.40) [− 6.90, 7.30]	106	1.72 (2.85) [− 9.10, 8.50]	106	0.08 (− 0.36,0.53)	0.71
1 s-NL	106	113.32 (7.70) [90.90, 130.90]	106	113.04 (6.27) [98.30, 127.20]	106	− 0.28 (− 1.31, 0.74)	0.59
1i-ML	106	95.75 (8.10) [70.40, 111.80]	106	93.12 (6.81) [63.40, 106.50]	106	− 2.64 (− 3.62, − 1.66)	< 0.001
1 s-1i	106	125.03 (10.35) [107.10, 151.60]	106	127.99 (7.80) [110.80, 153.60]	106	2.96 (1.65, 4.27)	< 0.001
SN-BA	106	131.58 (5.31) [112.10, 144.80]	106	131.78 (5.38) [111.40, 146.10]	106	0.20 (− 0.20, 0.59)	0.33
Upper incisors	106	10.91 (6.23) [− 5.00, 26.20]	106	12.23 (3.54) [3.90, 21.30]	106	1.32 (0.31, 2.34)	0.01
Lower incisors	106	5.45 (6.31) [− 13.40, 20.80]	106	0.21 (4.53) [− 18.90, 9.70]	106	− 5.24 (− 6.03, − 4.46)	< 0.001
Difference between achieved and nominal axial angulation for upper incisors			106	4.77 (3.54) [− 4.30, 13.10]			< 0.001
Difference between achieved and nominal axial angulation for lower incisors			106	− 6.21 (4.53) [− 15.70, 12.90]			< 0.001

*CI*, confidence interval; *SD*, standard deviation

**Table 3:** Linear regression of factors influencing the change in axial angulation for the upper or lower incisors through treatment (T1–T0)

		Δ upper incisor angulation			Δ lower incisor angulation		
Factor	Category	*b*	95% *CI*	*P*	*b*	95% *CI*	*P*
Initial tooth angulation	Per	− 0.52	− 0.63, − 0.41	** < 0.001**	− 0.38	− 0.48, − 0.28	** < 0.001**
Age	Per year	− 0.10	− 0.27, 0.08	0.28	− 0.19	− 0.42, 0.04	0.11
Gender	Female	Ref			Ref		
	Male	− 0.24	− 1.20, 0.72	0.62	0.39	− 0.46, 1.23	0.37
Vertical configuration	Mesofacial	Ref			Ref		
	Brachyfacial	− 0.32	− 1.41, 0.78	0.57	− 0.06	− 1.17, 1.06	0.92
	Dolichofacial	0.31	− 0.85, 1.46	0.61	− 0.13	− 1.66, 1.39	0.86
Angle classification	Class I	Ref			Ref		
	Class II/1	− 2.09	− 3.72, − 0.47	**0.01**	− 0.35	− 1.88, 1.18	0.65
	Class II/2	3.27	1.86, 4.68	** < 0.001**	− 0.13	− 1.61, 1.34	0.86
	Class III	0.98	− 0.28, 2.24	0.13	0.22	− 1.06, 1.51	0.73
Bracket type	Metal	Ref			Ref		
	Ceramic	− 0.18	− 1.10, 0.74	0.70	− 0.10	− 1.02, 0.81	0.82
Race	Caucasian	Ref			Ref		
	Oriental	− 0.09	− 1.13, 0.95	0.87	− 0.23	− 1.22, 0.77	0.65
	Japanese	0.03	− 4.25, 4.31	0.99	− 0.36	− 2.85, 2.12	0.77
Extraction	No	Ref			Ref		
	Yes	− 0.60	− 1.93, 0.73	0.38	− 1.25	− 2.45, − 0.06	**0.04**
SNA at T0	Per	− 0.06	− 0.21, 0.09	0.44	NT		
SNB at T0	Per	NT			− 0.04	− 0.21, 0.13	0.63
ANB at T0	Per	− 0.24	− 0.56, 0.09	0.16	− 0.07	− 0.38, 0.24	0.67
ΔANB T1–T0	Per	0	− 0.56, 0.57	0.99	− 0.17	− 0.65, 0.31	0.48
WITS at T0	Per mm	0.29	0.01, 0.57	0.04	0.24	− 0.10, 0.57	0.16
ΔWITS T1–T0	Per mm	− 0.23	− 0.51, 0.06	0.12	0.57	0.34, 0.79	** < 0.001**
Δ1s-NL T1–T0	Per	0.17	0.09, 0.26	** < 0.001**	NT		
Δ1i-ML T1–T0	Per	NT			0.40	0.28, 0.50	** < 0.001**

*b*, unstandardized regression coefficient; *CI*, confidence interval; *NT*, not tested; *Ref*, reference

*p* <0.05 is defined as significant

**Table 4:** Axial angulation of the upper incisors depending on ANGLE CLASS at baseline (T0), post-treatment (T1), and treatment-related changes (T1–T0)

Group	T0 Mean (SD)	T1 Mean (SD)	Delta T1–T0 Mean (SD)
Overall (*n* = 106)	10.9° (6.2°)	12.2° (3.5°)	1.3° (5.4°)
Class I (*n* = 23)	11.5° (5.8°)	11.7° (2.9°)	0.1° (4.7°)
Class II/1 (*n* = 19)	13.5° (4.9°)	10.5° (3.1°)	-3.0° (3.8°)
Class II/2 (*n* = 28)	4.8° (4.0°)	12.3° (3.2°)	7.5° (1.9°)
Class III (*n* = 36)	13.9° (5.2°)	13.5° (4.0°)	− 0.4° (4.2°)
*P* value*	< 0.001	0.02	0.07

^*^From generalized linear regression modelling

*SD*, standard deviation

**Table 5:** Axial angulation of the lower incisors depending on angle class at baseline (T0), post-treatment (T1), and treatment-related changes (T1–T0)

Group	T0 Mean (SD)	T1 Mean (SD)	Delta T1–T0 Mean (SD)
Overall (*n* = 106)	5.5° (6.3°)	0.2° (4.5°)	− 5.2° (4.1°)
Class I (*n* = 23)	5.7° (5.9°)	0.3° (4.0°)	− 5.3° (4.4°)
Class II/1 (*n* = 19)	9.3° (5.0°)	2.6° (3.8°)	− 6.7° (3.4°)
Class II/2 (*n* = 28)	5.4° (7.2°)	− 0.3° (4.4°)	− 5.6° (4.8°)
Class III (*n* = 36)	3.3° (5.7°)	− 0.8° (4.9°)	− 4.1° (3.6°)
*P* value*	0.007	0.05	0.59

^*^From generalized linear regression modelling

*SD*, standard deviation

**Fig. 3:**
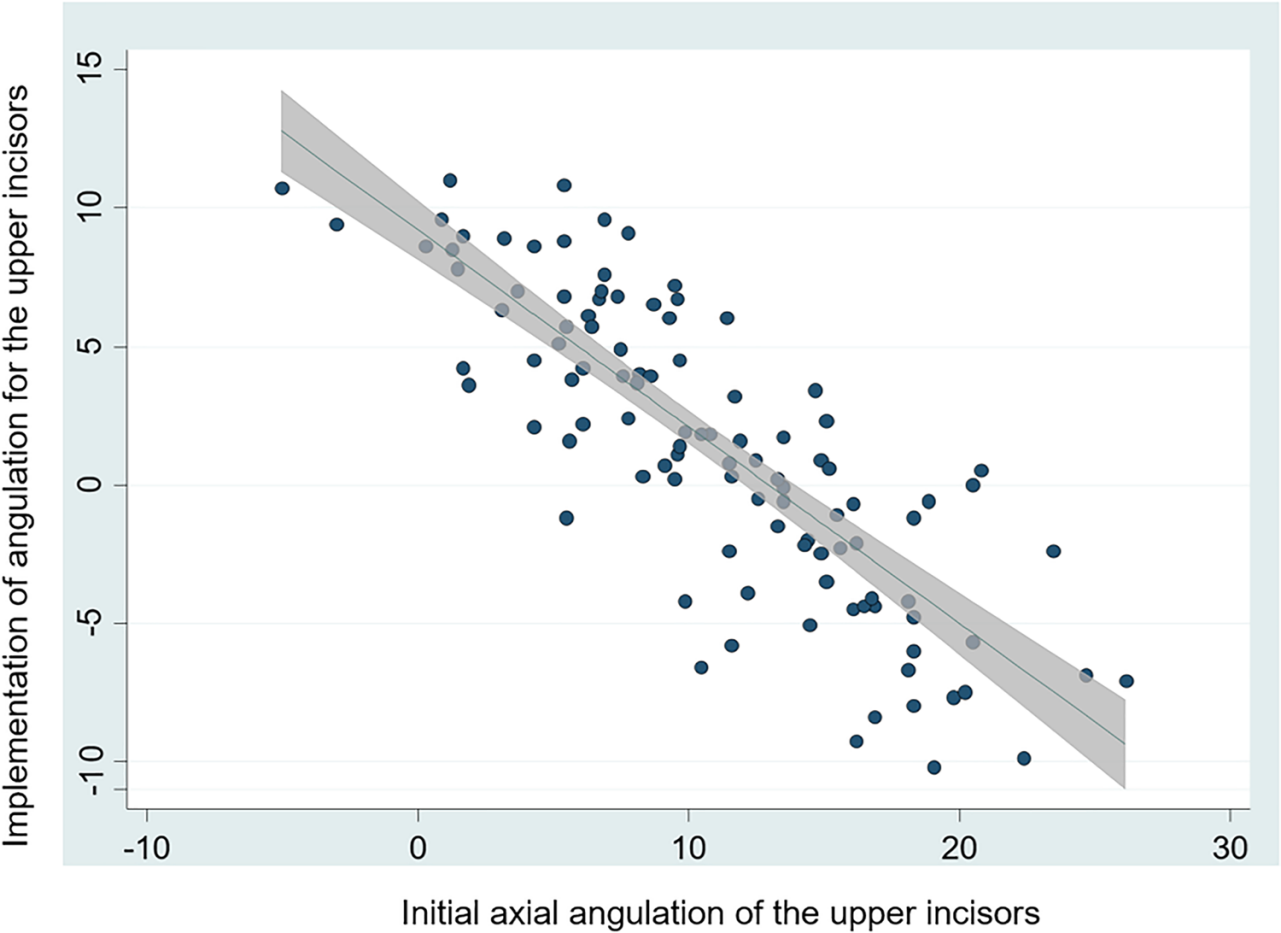
Final axial angulation of the upper incisors during treatment (*Y*-axis) as a function of the initial axial angulation (*X*-axis)

**Fig. 4:**
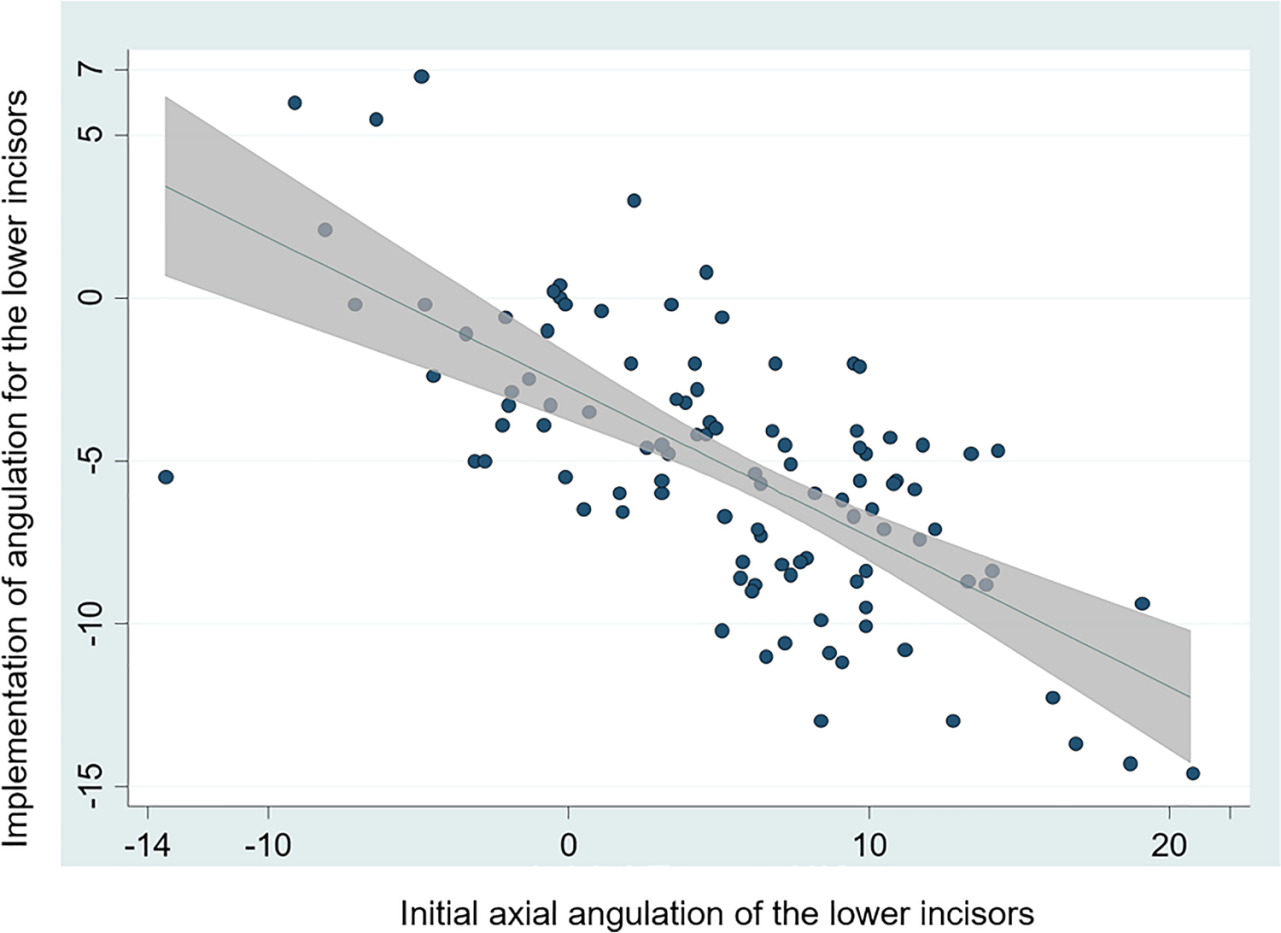
Final axial angulation of the lower incisors during treatment (*Y*-axis) as a function of the initial axial angulation (*X*-axis)

Table 3 lists the influence of all possible factors on treatment-related changes in the incisors’ axial angulation. First, initial axial angulations were significantly associated with treatment-related changes for both the upper and the lower incisors (*p* < 0.001 in both cases), which is graphically presented in Figs. 3 and 4. Greater axial angulation was transferred to teeth with initially smaller angulation, while teeth with greater angulation experienced smaller treatment-related changes. For the upper incisors, every 0.17° of greater angulation accounted for additional 1° of upper incisor proclination during treatment.

For the upper incisors, the parameters angle’s classification, baseline WITS, and treatment-related pro/retro-clination were associated with the transferred axial angulation during treatment (Table 3). Compared to Class I patients, Class II/1 patients experienced smaller (− 2.09°) and Class II/2 patients experienced greater treatment induced changes (+ 3.27°), while no difference was found for Class III patients. This might be explained with more proclined or retroclined incisors among Class II/1 or Class II/2 patients, respectively. Furthermore, baseline WITS was significantly associated with axial angulation changes, with 0.29° greater change for each additional mm in baseline WITS.

As far as the lower incisors are concerned, the parameters tooth extractions, treatment-related changes in WITS, and lower incisor pro/retro-clination were associated with treatment related changes in lower incisor axial angulation (Table 3). Extraction cases showed significantly smaller axial angulation transmission than non-extraction cases (-1.25◦), which might be explained by retraction of the lower incisors. WITS changes were associated with axial angulation changes, with 0.57◦ more angulation change for each additional mm increase in WITS during treatment.

Finally, angle’s classification was significantly associated with the axial angulation change of both upper (Table 4) and lower incisors (Table 5). For the upper incisors, significant differences according to the angle class were already found at baseline, with Class II/2 cases having significantly less angulated incisors compared to the other Classes. Treatment-related changes in upper incisor angulation likewise differed among classes, with Class I and Class III patients experiencing small changes (0.1° and − 0.4°, respectively), a small decrease for Class II/1 patients (− 3.0°) and a greater increase for Class II/2 patients (+ 7.5°). Lower incisor baseline axial angulation was likewise significantly different according to angle’s classification, with Class II/1 patients having more angulated lower incisors compared to other classes. However, in contrast to the treatment-related changes of upper incisors, relatively similar changes in lower incisor axial angulation were seen for all classes.

## Discussion

The present study investigated the implementation of the intended axial angulation of incisors in clinical routine and in addition determined whether certain initial morphological peculiarities or therapeutic measures were relevant for the implementation of this angulation in all available patients from a private practice treated within a defined time period. Thus, this study may be looked upon to contribute to the important topic of quality of care offering information about the outcome of orthodontic treatment in a routine clinical practice setting.

It was observed that during the observed therapy period of 38.94 weeks on average, the MBT prescription for the upper central incisors (+ 17°) was rarely implemented (9.4% of the cases) and for the lower incisors (− 6°) in no cases. The reasons for this may be multifactorial. Tooth movement in the form of targeted change of the axial angulation of the roots is one of the most complex and time-consuming aspects in orthodontics. The patient group examined finally received a 0.019 × 0.025-in stainless steel arch in a 22-slot system. In many practices, this arch dimension is routinely used as the last archwire for the 22-slot system. It provides a compromise between a larger wire that would be difficult to place and increase friction problems and on the other hand a smaller wire that delivers less tooth control. Theoretical calculation and in vitro studies have shown that the torque play in this constellation varies between 10 and 15°, depending on the bracket type and prescription [4, 6, 35, 41]. Thus, it might be possible that if the patients had been treated with a full-sized 0.021 × 0.025-in archwire with a greater potential for full torque expression, treatment may have resulted in less deviation from the nominal bracket prescriptions. However, as it was outlined before, the decision to finish treatment was in the hand of the attending orthodontist and our study aimed to reproduce clinical practice reality as close as possible.

Today, some clinicians suggest to apply individualized torque prescriptions based on the treatment needs of the individual patient [22]. Taking into account the slot play of the final wire, the orthodontist could pick an optimal bracket wire combination that would move every tooth to its desired position without the necessity of individual archwire torque adjustments. In addition, with the help of computerized fabrication techniques, individual torque prescription can be integrated into the slot of a custom fabricated bracket for every tooth or it may be bent into a custom prefabricated archwire. These “fully customized appliances” typically use slot-filling wires and are based on a setup as a treatment objective. On the other hand, up to now, convincing evidence documenting that these advanced techniques might result in superior treatment results is missing [9, 28, 38].

Transmission of labio-lingual angulation is also influenced by other factors such as the material properties of the archwire, the type of ligature, and the interbracket distance [4, 21, 29, 30]. Further on, the duration of moment application plays an important role for the conversion of axial angulation change. In this study, the last working archwire (0.019 × 0.025-in stainless steel) was left in place for approximately 8 weeks at the mean. It may be worth a discussion, whether this duration was sufficient to achieve adequate treatment effects in all patients. Nevertheless, the time point to finish this phase of treatment was individually chosen by the orthodontists on the basis of subjective evaluation and according to clinical experience.

In our study, the angle class to start with showed a significant influence on the implementation of axial angulation changes of the incisors. Individual variation in axial angulation of incisors was repeatedly reported and was interpreted to express compensation in cases of sagittal discrepancy [7, 18, 43]. In our study, we observed that larger treatment induced changes in the maxillary incisors in angle Class II compared to angle Class I or angle Class III cases. In contrary, treatment-induced angulation changes in the mandibular incisors were relatively similar in all malocclusions. This is in accordance with a study published by Bakos [6], who in 2015 found that the greatest axial angulation changes in maxillary incisors occurred in Class II/2 malocclusion. We found that the initial angulation for the upper incisors (protrusion) was greatest in angle Class III malocclusion at T0 and still at T1. This result may as well be explained by the dento-alveolar compensation mechanism [43] which is typical for Class III malocclusion cases.

As expected, it was found that the smaller the initial angulation, the greater the change, and the closer it was to the prescription, the less change took place. It is important to mention here that the achievement of the defined prescription must be planned individually in each individual case. In some cases — as for example in Class III cases — it is often mandatory not to fully implement the prescription for positioning the lower incisors.

Earlier studies have already shown that the vertical skeletal configuration also plays an essential role for the position of the incisors. In their cephalometric investigations, Hasund and Ulstein [18] were able to demonstrate that the face type had a significant influence on the angulation of the incisors. Similarly, it was found out that the vertical skeletal configuration expressed by the mandibular plane angle (ML-NL) plays an important role for the position of the incisors [10, 32]. In our study, we defined the vertical skeletal configuration by the mandibular plane angle, too. We observed a tendency towards an axial angulation changes greater than the preprogrammed values for the upper as well as for the lower incisors in cases with a dolichofacial configuration compared to patients with a horizontal one although this difference did not reach statistical significance.

As stated above, treatment-induced axial angulation changes to a large extent depend on the torque-play and the type of archwire alloy [11, 15, 17]. In our study, about one-half of the patients were treated with conventional metallic brackets (Victory—3 M Unitek) while the other half received self-ligating ceramic brackets (In-Ovation C—GAC). Statistical group comparison did not demonstrate any significant difference between these two bracket types with respect to treatment induced axial angulation changes in our patients. Concerning the any influence of the self-ligating mechanism, one must distinguish between active and passive systems. Recent literature evaluated that passive self-ligating systems were associated with lower conversion of axial angulation [21]. In a study by Katsikogianni et al. [23], active self-ligating brackets showed the best transmission of axial angulation; however, this study was performed in vitro. In our clinical study, where an active variant of self-ligation was applied, the type of bracket and the type of ligation would not be of any significant influence.

## Limitations of the study

There are some shortcomings in our study. First of all, it is its retrospective design, which typically introduces different kinds of bias. According to Papageorgiou et al. [37], due to frequent selection and observation bias, retrospective studies typically result in inflated treatment effects compared with prospective ones. However, owing to a primary focus of this study on quality care in routine clinical practice, selection of cases was made from all patients of one clinical practice being treated within a defined time frame. No pre-calculation of a sample size was performed. The further above-presented post-hoc analyses which were run as a compensation are typically calculated on negative trials [25]. As an alternative to post-hoc power analysis, the width and magnitude of the 95% confidence interval (95% *CI*) were suggested to be a more appropriate method [25]. Doing so, in our study, the 95% confidence intervals (95% *CI*) for the mentioned deviations of the achieved axial angulation of the upper and lower incisors from their respective nominal torque values at T1 were calculated to be 4.09° < 4.77° (mean) > 5.44° (*p* < 0.001) for the upper incisors and − 7.07° <  − 6.21° (mean) >  − 5.34° (*p* < 0.001) for the lower ones (see additional data in Table 2). These data enhance the confidence in the received statistical results and may be of help if planning future studies on the topic and performing pre-calculation of adequate sample size.

In the present study, two different kinds of brackets were used (conventionally ligated metal or self-ligating ceramic brackets) but were both of an MBT prescription with the same pre-programmed torque values for central and lateral incisors. However, these two bracket systems were manufactured from two different companies and as a result might have undergone quality management procedures of varying diligence. This might have an impact on their ability to transfer adequately applied moments on the teeth, even though no statistically significant differences in torque were found between the two systems (Table 3). Furthermore, the treatment plan for each patient (including tooth extractions, ligatures, or elastics, power chains) was left to the discretion of the treating orthodontist based on what was needed to achieve the optimal treatment results. However, the large sample size of the present study and the incorporation of such factors in the statistical analysis minimize the potential impact of these factors on the observed results.

Analysis of axial angulation on the cephalograms may have been another source of error. The limitations and errors of two-dimensional cephalometry have been widely discussed [12, 45, 49]. Instead of using conventional cephalometric reference planes, in our study, axial angulations of upper and lower incisors were assessed between a perpendicular to the archwire plane and a line representing the bracket base of the mostly anterior positioned incisors. In his original prescription, Andrews defined axial bracket angulation in relation to a perpendicular to the occlusal plane, which he defined as a plane passing through the occlusal surfaces of the teeth [1, 2]. In addition, Andrews defined another reference plane created by the centers of each clinical crown (LA-points) and which he called “Andrews plane.” One plane was defined for the mandible and one for the maxilla. These planes should ideally be parallel with each other and also with the occlusal plane. They are also regarded as representing the “bracket positioning plane” [1, 2, 50] and thus should coincide with the plane of the treatment archwires being ligated into the brackets which we chose as the reference for our measurements [13]. Although intra-examiner reliability in our study was determined to be high, alternative methods like measurements on plaster casts with or without 3D-scanning or direct analysis of intraoral scans have been documented to be a good alternative avoiding exposition to x-rays and being possibly even more precise than lateral radiographs for this purpose [12, 24, 45, 49]. In addition, due to the nonexistence of really stable intra-maxillary reference systems in growing patients, it was not possible to differentiate between real torque movements according to the definition mentioned above and changes of angulation of the incisors brought about by a combination of root and tooth movements.

## Conclusions

Analyzing a daily practice orthodontic treatment sample, the available data revealed clinically relevant factors for the transmission of axial angulation changes of upper and lower incisors:

- The initial angulation value was the mostly relevant factor.
- The angle class also had an influence on the conversion of intended angulation changes.
- The vertical skeletal configuration and type of treatment (extraction vs. non-extraction) should also be considered.
- The bracket type appeared less relevant in our study for the implementation of the intended changes.

In daily practice, it is therefore mandatory for those orthodontists who use a single prescription setup of brackets to carefully evaluate tooth positions and especially axial angulations of the incisors in the final stage of treatment. Individualized finishing bends may have to be placed in order to achieve the desired treatment goal.
